# Malaria prevention knowledge, attitudes, and practices in Zambezia Province, Mozambique

**DOI:** 10.1186/s12936-021-03825-9

**Published:** 2021-06-30

**Authors:** Liliana de Sousa Pinto, Jorge A. H. Arroz, Maria do Rosário O. Martins, Zulmira Hartz, Nuria Negrao, Victor Muchanga, Amadeu Cossa, Rose Zulliger

**Affiliations:** 1 Mozambique Medical Coucil, Maputo, Mozambique; 2 Global Health and Tropical Medicine, GHTM, Instituto de Higiene E Medicina Tropical, IHMT, Universidade Nova de Lisboa, UNL, Rua da Junqueira 100, 1349-008 Lisboa, Portugal; 3 Cactus Communications, A/603, Satellite Gazebo, Guru Hargovindji Marg, Andheri East, Mumbai, 400093 Maharashtra India; 4 Plataforma Inter-Religiosa de Comunicação para Saúde–PIRCOM, Maputo, Mozambique; 5 Independent Consultant, Maputo, Mozambique

**Keywords:** Knowledge, Attitude, Practices, Mozambique, Malaria, Social and behaviour change

## Abstract

**Background:**

In Mozambique, socio-economic and cultural factors influence the wide adoption of disease preventive measures that are relevant for malaria control strategies to promote early recognition of disease, prompt seeking of medical care, sleeping under insecticide-treated nets (ITNs), and taking intermittent preventive treatment for pregnant women. However, there is a critical information gap regarding previous and ongoing malaria social and behavioural change (SBC) interventions. The aim of this study is to assess the knowledge, attitudes, practices of beneficiaries of SBC interventions.

**Methods:**

A descriptive cross-sectional survey was undertaken in 2018 in two rural districts of Zambezia Province, Mozambique. A structured questionnaire was administered to 773 randomly selected households. Respondents were the adult heads of the households. Descriptive statistics were done.

**Results:**

The main results show that 96.4% of respondents recalled hearing about malaria in the previous 6 months, 90.0% had knowledge of malaria prevention, and 70.0% of preventive measures. Of the 97.7% respondents that had received ITNs through a mass ITN distribution campaign, 81.7% had slept under an ITN the night before the survey. In terms of source of health information, 70.5% mentioned the role of community volunteers in dissemination of malaria prevention messages, 76.1% of respondents considered worship places (churches and mosques) to be the main places where they heard key malaria prevention messages, and 79.1% asserted that community dialogue sessions helped them better understand how to prevent malaria.

**Conclusions:**

Results show that volunteers/activists/teachers played an important role in dissemination of key malaria prevention messages, which brought the following successes: community actors are recognized and people have knowledge of malaria transmission, signs and symptoms, preventive measures, and where to get treatment. There is, however, room for improvement on SBC messaging regarding some malaria symptoms (anaemia and convulsions) and operational research is needed to ascertain the drivers of malaria prevalence and inform the SBC approach.

## Background

In 2018, there were an estimated 228 million cases of malaria globally, the vast majority (93%) in the African region [1]; Mozambique is one of the six countries to account for more than half of all malaria cases. It is important to understand the factors that contribute to such a high disease burden in the country.

The World Health Organization’s Global Technical Strategy for Malaria 2016–2030 is comprised of three major pillars with two supporting elements: (i) innovation and research, and, (ii) a strong enabling environment [2]. These supporting elements are aligned with the Mozambican National Malaria Control Programme (NMCP) for the elimination of malaria through implementation of research to optimize the impact and cost-effectiveness of new and existing tools, interventions and strategies, strong political and financial commitments, multi-sectorial approaches, stewardship of the health system, and capacity building development [3].

The NMCP prioritizes planning, implementation, monitoring, and evaluation based on an evidence-based, multi-cultural, and gender equality approach and an interpersonal communication and mass media (radio) approach [3]. However, there is a critical information gap about outcomes and impact of social and behavioural change (SBC) interventions in Mozambique [3]. SBC interventions are widely used in malaria prevention and control programmes to promote appropriate care-seeking and provision and utilization of insecticide-treated nets (ITNs) and indoor residual spraying (IRS). These interventions play an important role in increasing knowledge and creating awareness and the demand for prevention and treatment programmes [4]. Human behaviour is an important factor contributing to disease burden [5]. It is important to do formative research into various aspects that influence human behaviour, such as individual preferences, community characteristics, leadership practices, and quality of available goods and services, to determine their impact and to design effective SBC strategies and interventions [5]. Behavioural research is also useful for evaluation of SBC interventions.

In Mozambique, the goal of research on SBC interventions is to identify knowledge, attitudes, practices (KAP), and behaviours of communities, in order to define key strategies, target groups, cultural barriers, and community beliefs for improving malaria health outcomes through the adoption of positive health behaviours [3, 6]. This finding is similar to a study conducted in rural Tanzania which demonstrated that more research on malaria knowledge and beliefs of the community is necessary to obtain and maintain community engagement and participation in malaria control activities [7]. The importance of obtaining this knowledge was underlined in a study conducted in Southeastern Iran that showed that strategies for the control of malaria can be effective, useful and valuable if prior studies are taken to explore and understand people’s KAP [8]. In addition, and encouragingly, a study from rural Uganda indicated that communities with knowledge can influence practices in households and support control of the disease [9]. In areas with high burden of disease it is important to have a clear understanding of the community to design good SBC interventions.

The present study was conducted with the aim of assessing the KAP of beneficiaries of malaria prevention SBC interventions in rural Mozambique.

## Methods

### Study area and design

This study was a cross-sectional survey carried out in November and December 2018 in Namacurra and Nicoadala districts of Zambézia Province. The districts were selected based on: (i) high malaria incidence; (ii) accessibility; (iii) population size similarities; (iv) geographic location; and, (v) experience with SBC interventions. The estimated population of Namacurra and Nicoadala is 390,410 and 270,825 inhabitants, respectively [10]. Both are rural districts with more than 60% of the population being illiterate and living in low social and economic conditions. The main public health problems are: malaria, HIV and diarrhoeal diseases [11]. In 2017, the incidence of malaria (per 1000 inhabitants) in Namacurra was 272 and in Nicoadala was 506 [12]. Both districts have targeted SBC and vector control interventions. SBC interventions included training of volunteers, local religious and community leaders, members of community structures, schoolteachers, and community health workers. These are all community actors that play an important role in dissemination of malaria prevention key messages. Additionally, different communication channels used to reach community members by these actors include meetings with health committee councils and health facilities, dissemination of standardized malaria SBC messages through community radios, door to door visits, sermons at worship places (mosque and church), community dialogues, focus groups discussions with adults, and dissemination of information, education and communication materials. Health committee councils, focal groups discussions (men to men, women), and community dialogues are part of the Mozambique National Health Promotion Strategy to promote and protect individual, family and community health by promoting positive health behaviours. Previously validated community dialogues are also used [13]. Community dialogues to promote healthy living habits are based on a set of ‘Life Stories’ prepared and used in a series of sessions to stimulate dialogue between people living in the same geographic area (neighbourhoods or communities). Additionally, women and men are given tools that enable them to reflect on how gender norms and social roles work in their lives, and the skills to begin a process of changing those norms, beliefs and roles that are considered harmful to health and the environment, and to the social well-being of people and communities, while reinforcing those that are perceived as positive and to be maintained [13].

Many of these efforts, particularly promotion on net use, were connected to the ITN universal coverage campaign (UCC) distribution, which covered the population of each district in 2017, and through ITN distribution in antenatal care (ANC) services to pregnant women. UCC in 2017 distributed 161,591 ITNs in Namacurra (100% of target) and 125,161 ITNs in Nicoadala (86% of target) [14].

All localities of these districts were selected for the study. Within each locality, household sample size was calculated by dividing the total sample size of the district by the number of existing localities. Households, the sampling units used in this study, were selected using a systematic random sampling method, after determining the total number in each locality.

### Sample size

Sample size was calculated based on the equation:

1 $$n={Z}^{2}\times p\times (1-p)/d$$

where: n = sample size; Z = 1.96 (assuming a level of confidence of 95%); p = proportion = 0.5; d = error = 0.05. A total of 768 households were required for the study, 384 per district. The households were divided between localities with sample size equal to the proportion of households per locality. Additionally, five households were added during data collection, resulting in a study population of 773 households.

### Selection of households

In each locality, the households were selected based on the following strategy: first, a household list (sampling frame) was developed and a number was assigned to each household; then, the sample interval (number of households divided by sample size) was computed and a random start number was chosen; finally, from this first random number, households were systematically selected using the sampling interval until the calculated sample size was met.

### Data collection and measurement

A structured, close-ended questionnaire was pre-tested and administered by previously trained local interviewers. The first section of the questionnaire included standardized socio-demographic questions based on the Malaria Indicator Survey 2018 and the following parts of the questionnaire assessed the head of the household malaria KAP/behaviours, and information channels. The questionnaire was designed in Portuguese, and the interviews were conducted in the local language, *Enlowe*. The questionnaire was pre-tested in a district similar to the study districts. The head of the household was defined as the primary decision-maker in the family and the household and as an individual living in the household and having meals from a common cooking facility [15]. A responsible adult, 18-years or older, was appointed to participate in the interview in the absence of the head of the household.

### Variables

The variables selected for this study were: place of residence, age, gender, level of education, number of people that live in the household, information channels, malaria KAP.

### Household inclusion criteria

The inclusion criteria used to select the households for the study were: (i) households from the selected districts; (ii) household members living in the district from 2011 to 2017 (this period covers the SBC interventions funded by different malaria donors); (iii) interviewee at least 18 years old (head of the household), regardless of gender; (iv) community located in the study districts in which SBC interventions were performed by local community actors (volunteers from community structures, schoolteacher facilitators, activists, and faith leaders); (v) mosquito net mass distribution campaigns; and, (vi) presence of community radios.

### Outcomes of interest

The measured outcomes were: (i) percentage of people who remember hearing or seeing a message about malaria in the previous 6 months; (ii) percentage of people with favourable attitudes towards ITNs, malaria-related practices (use of ITNs, taking anti-malarials) and services (timely demand for health, institutional or community services when noticing signs and symptoms of malaria); (iii) percentage of people who believe the majority of their friends and communities practice the behaviours (using ITNs and seeking counselling and health care services); (iv) percentage of people who identify the mosquito as a cause/vector of malaria; (v) percentage of people who recognize the main signs and symptoms of malaria; (vi) percentage of people who know about treatment for malaria; (vii) percentage of people who know malaria prevention measures; (viii) percentage of households with at least one ITN; (ix) percentage of households with one ITN for every 2 people; (x) percentage of people with access to mosquito nets; xi) percentage of people who slept under an ITN the night before the survey; and, xii) use/access ratio of mosquito nets: behaviour indicator.

### Data and statistical analysis

After conducting the study, the previously coded questionnaires were reviewed to verify the responses and their validation; later, the data were entered in SPSS for Windows, version 23.0 (IBM; Armonk, NY, USA). Data analysis was based on descriptive and inferential statistical analysis,

## Results

### Sociodemographic characteristics of participants

A total of 773 household heads were interviewed of whom 59% were females and 41% males (Table 1). The mean age was 34.6 years (range 18–90 years; standard deviation: 7.7). About 27.4% were illiterate, 63% had completed primary school, and 8.4% had basic education. About half of the respondents (49.9%) lived in households with 4 to 6 people, a quarter (26.3%) in households composed of 1 to 3 members, and the remainder in households with 7 members or more (23.8%). Detailed socio-demographic characteristics are presented in Table 1.

**Table 1:** Sociodemographic characteristics of respondents in the selected households from Nicoadala and Namacurra

Characteristics	N = 773	Percentage (%)
Household size		
1 to 3	206	26.6
4 to 6	385	49.8
≥ 7	182	23.5
Total	773	100.0
Household members frequently present in the last 6 months		
1 to 3	203	26.3
4 to 6	386	49.9
≥ 7	184	23.8
Total	773	100.0
Gender		
Male	317	41.0
Female	456	59.0
Total	773	100.0
Age (years)		
< 18	26	3.4
18 to 24	209	27.0
25 to 34	175	22.6
35 to 49	206	26.6
50 to 64	114	14.7
65+	27	3.5
Unknown	16	2.1
Total	773	100.0
Level of education completed		
No education	212	27.4
Primary and elementary	487	63.0
Basic	65	8.4
Secondary	8	1.0
Tertiary and university	1	0.1
Total	773	100.0

### Knowledge regarding malaria prevention and treatment

Table 2 shows that within 773 household heads, about 96% reported having heard and 3.3% never having heard about malaria. However, the majority of respondents (96.4%), recalled hearing about malaria in the previous 6 months.

**Table 2:** Reported exposure to information about malaria

Variable	N	Percentage (%)
Ever heard of malaria?		
Yes	695	96
No	26	3.3
Without answer	52	6.7
Total	773	100.0%
If yes, any exposure in the previous 6 months?		
Yes	695	100
No	0	0
Total	695	100.0

Most respondents (83.4%) reported that malaria is transmitted through mosquito bites. Regarding recognition of malaria symptoms, headache was pointed to as one of the main symptoms of malaria by both male (48.9%) and female (48.7%) respondents. Body pain was the second most mentioned symptom by 39.1% of males and 34.2% of females. About 3.9% of respondents were unable to identify any symptoms of malaria. In Table 3, it can be noted that the most frequently reported malaria preventive measure was the use of ITNs (72.2%). More men (76%) reported the use of an ITN than women (70%), burning garbage and creating smoke to chase away mosquitoes (35%) and improving the cleanliness and hygiene of house and yard (24%) were other preventive measures mentioned by more than a fifth of respondents. IRS was one of the least mentioned forms of prevention (4.3%). The use of insecticide products and repellents were rarely mentioned, 3.1% and 1.8%, respectively.

**Table 3:** Level of knowledge about malaria

Type of Knowledge	Male		Female		OR	95% CI	Total	
	N = 317	(%)	N = 456	(%)			N = 773	(%)
Reported cause of malaria transmission								
Mosquito bite	271	85.5	374	83.4	1.68	1.20–2.34*	645	83.4
Garbage/dirt near the house	84	26.5	135	29.6	1.17	0.84–1.61	219	28.3
Others (fleas/lice)	49	15.5	67	14.7	0.94	0.63–1.41	116	15.0
Does not know	17	5.4	31	6.8	1.28	0.70–2.36	48	6.2
Recognition of symptoms								
Body pain	124	39.1	156	34.2	0.81	0.60–1.08	280	36.2
Headaches	155	48.9	222	48.7	0.99	0.74–1.32	377	48.8
Joint pain	74	23.3	133	29.2	1.36	0.97–1.88*	207	26.8
Diarrhoea	39	12.3	86	18.9	1.66	1.10–2.49*	125	16.2
Vomit	46	14.5	114	25.0	1.96	1.34–2.86*	160	20.7
Lack of appetite	49	15.5	80	17.5	1.16	0.79–1.71*	129	16.7
Cough	5	1.6	7	1.5	0.97	0.30–3.09	12	1.6
Nasal congestion	1	0.3	1	0.2	0.69	0.04–11.1	2	0.3
Does not know	9	2.8	21	4.6	1.65	0.74–3.65	30	3.9
Reported malaria preventive measures								
Burn leaves/eucalyptus	14	4.4	40	8.8	2.08	1.12–3.89*	54	7.0
Insecticide serpentine/spray	4	1.3	20	4.4	3.63	1.23–10.7*	24	3.1
Mosquito net	241	76.0	319	70.0	0.73	0.53–1.01*	558	72.2
Repellent	3	0.9	11	2.4	2.59	0.71–9.35	14	1.8
Burn garbage	112	35.3	156	34.2	0.95	0.70–1.28	268	34.7
Traditional treatment	3	0.9	6	1.3%	1.39	0.34–5.62	9	1.2
Improve home hygiene	79	24.9	109	23.9	0.95	0.67–1.32	188	24.3
Spraying/fumigate the house	8	2.5%	25	5.5	2.24	0,99–5.30*	33	4.3
Improve individual hygiene	16	5.0%	15	3.3	0.65	0.31–1.31	31	4.0
None/does not know	23	7.3%	42	9.2	1.29	0.76–2.20	65	8.4

*OR* odds ratio, *CI* confidence interval

* Significant association (p < 0.05)

### Practices of malaria prevention, ownership and use of bed nets in target communities

About 80% of respondents reported having at least one bed net hanging at home, 21% reported having only one, 37.2% having two, 26.7% having three, and 13.3% having four or more. Most of the respondents (97.7%) reported that they received bed nets through the mass universal coverage ITN distribution campaign, and 82.7% reported sleeping under the bed net the night before the survey (Tables 4 and 5).

**Table 4:** Treated bed net ownership

Variables	N	Percentage (%)
Households has at least one bed net hanging at home		
Yes	618	79.9
No	103	13.3
No answer	52	6.7
Total	773	100.0
Number of bed nets at home		
One bed net	131	21.2
Two bed net	230	37.2
Three bed net	165	26.7
Four bed net	53	8.6
Five or more	29	4.7
Don’t know	10	1.6
Total	618	100.0
Source of bed net		
Universal coverage campaign	604	97.7
Antenatal care	4	0.6
Don’t know	10	1.6
Other places	0	0.0
Total	618	100.0

**Table 5:** Bed net usage

Did you sleep under a bed net the night before	Male		Female				Total	
	Freq. (N = 301)	Perc. (%)	Freq. (N = 420)	Perc (%)	OR	95% CI	Freq. (N = 618)	Perc. (%)
Yes	224	86.8	287	79.7	0.55	0.35–0.85*	511	82.7
No	34	13.2	73	20.3			107	17.3

* p value 0.003

### Beneficiaries’ attitudes towards malaria prevention, diagnosis and treatment

From the 721 respondents that reported exposure to information about malaria, 86.3% felt confident about their knowledge of how to prevent malaria and 96.4% knew where to get treatment. Regarding the use of bed nets at night, 96.9% of respondents considered it an important prevention from malaria. Most of the respondents (69.2%) reported that family members, friends and neighbours influence their decision-making regarding their health and 23.6% disagreed with this assertion (Table 6).

**Table 6:** Attitudes of beneficiaries regarding malaria prevention

Variables	N	Percentage (%)
I feel confident that I know how to prevent malaria		
Disagree or strongly disagree	70	9.7
Neutral	29	4.0
Agree or strongly agree	622	86.3
Total	721	100.0
I know where I can get treatment for malaria		
Disagree or strongly disagree	16	2.2
Neutral	10	1.4
Agree or strongly agree	695	96.4
Total	721	100.0
The use of a bed net every night is important so that I can protect myself from getting malaria		
Disagree or strongly disagree	17	2.4
Neutral	5	0.7
Agree or strongly disagree	699	96.9
Total	721	100.0
My family, friends and neighbours influence my decision-making regarding my health		
Disagree or strongly disagree	170	23.6
Neutral	52	7.2
Agree or strongly agree	499	69.2
Total	721	100.0

Table 7 shows attitudes regarding measures adopted for malaria diagnosis and febrile symptoms. From the 721 heads of household that had heard of malaria, 539 (74.8%) reported a household member with a fever in the previous 6 months. In 409 households a family member was reported to have had a fever in the two weeks prior to the survey, among whom 395 reported seeking counselling and treatment from health facilities, 10 from the market, and 4 from other places. Participants reported that they sought care at the health facility services because it had better quality/was more efficient (66.1%), and was less expensive (22.0%). The person who decided where to seek counselling and treatment was generally the head of the household (72.9%), followed by the spouse of head of household (16%), then the person with fever (9.0%). From 395 households, 359 reported a rapid diagnostic test (RDT) was taken by a health worker and the remaining 39 were not tested. Of 359 respondents who were tested, 89.7% obtained a positive result, 0.8% negative, and 9.5% did not know their result.

**Table 7:** Beneficiaries' attitudes towards malaria diagnosis and treatment

Variables	N	Percentage (%)
Household member had a fever in the previous 6 months		
Yes	539	74.8
No	175	24.3
Does not know/does not remember	7	1.0
Total	721	100.0
Household member had a fever in the previous two weeks		
Yes	409	56.7
No	273	37.9
Does not know/Does not remember	5	0.7
Total	687	95.3
Did you look for someone for malaria treatment?		
Yes	409	100
No	0	0
Total	409	100
Where did you seek counselling and treatment?		
Health Facility	395	96.6
Market	10	2.4
Another place	4	1.0
Total	409	100.0
Reason sought advice or treatment from this provider		
More efficient/competent/better quality	261	66.1
Less expensive	87	22.0
Close to their residency	36	9.0
Other reason	11	3.0
Total	395	100.0
Who decided where you went to for counselling/treatment?		
Head of household	290	72.9
Wife of the head of household	67	16.8
Mother-in-law	1	0.3
The person with the fever	36	9.0
Other	4	1.0
Total	395	100.0
Reported use of malaria test		
Yes	359	90.2
No	39	9.8
Total	395	100.0
Malaria test result		
Positive	322	89.7
Negative	3	0.8
Do not know	34	9.5
Total	359	100.0

### Attitudes of beneficiaries regarding malaria prevention

As shown in Table 8, around 70.5% of respondents felt that community volunteers were ready to disseminate key malaria prevention messages. From 721 respondents, 76.1% identified worship places (churches and mosques) where they heard key messages on malaria prevention. Some 79.1% strongly agreed that community dialogue sessions helped them better understand how to prevent malaria. For the respondents, volunteers/activists/teachers played an important role in the dissemination of key malaria prevention messages.

**Table 8:** Attitudes of beneficiaries regarding malaria prevention

Variables	N		Percentage (%)
I feel that community volunteers are prepared to spread key malaria prevention messages			
Disagree or strongly disagree	86		11.9
Neutral	76		10.5
Agree or strongly agree	559		77.5
Total	721		100.0
Did you receive sufficient information from the activists?			
Disagree or strongly disagree	113		15.7
Neutral	102		14.1
Agree or strongly agree	506		70.2
Total	721		100.0
Most of my neighbours, my community and my family including myself have the ability to recognize the signs and symptoms of malaria			
Disagree or strongly disagree	90		12.5
Neutral	46		6.4
Agree or strongly agree	585		81.1
Total	721		100.0
Worship places (churches and mosques) are where I hear malaria prevention messages			
Disagree or strongly disagree		133	18.4
Neutral		39	5.4
Agree or strongly agree		549	76.1
Total		721	100.0
Community dialogue sessions helped me better understand how to prevent malaria			
Disagree or strongly disagree		65	9.0
Neutral		86	11.9
Agree or strongly agree		570	79.1
Total		721	100.0
Volunteers/activists/teachers play a key role in spreading key messages on malaria prevention			
Disagree or strongly disagree		55	7.6
Neutral		65	9.0
Agree or strongly agree		601	83.4
Total		721	100.0

## Discussion

Most malaria prevention strategies are centred on human behaviour and SBC interventions are a key part of the NMCP malaria strategy. This study assessed malaria prevention and treatment KAP in two rural Mozambican districts, Namacurra and Nicoadala.

The results show that almost all respondents had heard about malaria in the previous 6 months and those in these rural Zambezia districts have at least some knowledge of malaria causes, symptoms, treatment, and preventive measures. These results are similar to those obtained in other studies [7–9, 15], implying that the SBC campaigns of previous years have been successful at reaching people in rural Mozambique and somewhat successful at disseminating education messages. However, it is important to note that although most respondents knew that malaria is transmitted by the mosquito bite, they did not associate it with other people (i.e., with “bites of mosquito which bit a malarial patient”). This lack of knowledge has been reported before [8, 15], and is an indication that messaging on this aspect of transmission needs to be improved. Headaches were identified as the main symptom of malaria, similar to a study conducted by Khumbulani et al. [16]. However, despite relatively good knowledge of malaria symptoms and signs, respondents failed to name anaemia and convulsions. This lack of information could lead to a delay in seeking appropriate care from health facilities or community health workers. It is important to improve the training of local health community actors and subsequently improve dissemination and explanation to beneficiaries.

Most respondents felt confident and knew about malaria prevention methods and where to seek treatment, and considered the use of bed nets important to prevent and protect from malaria, which is similar to a study conducted in Ethiopia where the majority of respondents considered the mosquito net a protective measure against mosquito bites [17]. ITNs are a key part of malaria prevention strategies. ITNs are distributed through key channels, with most distributed through mass distribution campaigns and through antenatal care consultations [1]. In Mozambique 68% of the population sleeps under a bed net (40% under LLINs). These results show that previous SBC campaigns on bed nets have likely been successful in rural Zambezia as this were among the most recognized prevention form among the respondents, most of them having at least one bed net hanging at home which they use every night. Additionally, most reported sleeping under a bed net the night before the survey, which is similar to other studies conducted in the country [7, 15, 18].

Despite the wide availability of bed nets in the region and the indication from the results that the community uses them, the prevalence of malaria in Zambezia Province in children aged 6 to 59 months (using the malaria diagnostic test) increased from 38.3% in 2011 [19], 40.2% in 2015 [20] and 39% in 2018 [15]. Whilst efforts to support improved and regular use of nets by all is required, which may need more nuanced messaging for different audiences, other methods of vector control may also need to be investigated for a further reduction in prevalence.

In this study, health facilities were most commonly used for malaria treatment. This observation is similar to other studies [7, 21], and it was pointed out as being the more effective and less expensive place to go to. The decision about where to go to receive treatment was the responsibility of the head of the household. Data show that family members, friends and neighbours were the greatest influencers on decision-makers regarding health of members of the household, similar to a study conducted in Nigeria, where it was indicated that family members play a role on health decisions [22]. This reinforces the importance of having strategies and approaches in SBC to target greatest influencers.

The majority of respondents consider community volunteers/activists/teachers are very well trained and they play an important role in disseminating key malaria preventive messages. These findings are collaborated by a study conducted in Kenya which showed that community actors are very well accepted during community implementation of SBC interventions [23]. Worship places (churches and mosques) are where respondents heard malaria preventive messages, and community dialogue sessions helped them better understand how to prevent malaria. Similarly, in a study conducted in Nampula Province, Mozambique, respondents affirmed that community dialogues helped communication and ultimately encouraged malaria-related behaviour [24]. These results indicate that the people implementing SBC interventions in Mozambique aid in dissemination of information and are accepted interlocutors by the beneficiaries.

This study has some limitations: it targeted the head of household as a proxy to KAP held by all members of a household. Ideally, a broader sampling method across the range of adults within Nicoadala and Namacurra communities should have been used. However, this was not possible due to funding constraints. The results may not accurately represent the community’s perspectives as a whole. Another limitation of the study is that people in these communities could have obtained information from sources other than the formal SBC interventions, e.g., social media or informal conversations. One cannot attribute all of the success to previous formal SBC interventions. All reported behaviours were self-reported and may have been affected by social desirability bias. Additionally, the questionnaire was not designed to document net quality. Low net quality might have influenced the effectiveness of the intervention, as noted in other studies from Mozambique [25].

## Conclusions

This study confirms that SBC interventions carried out in rural Mozambique have had many successes. Namely, most people have knowledge of malaria prevention and treatment. The study shows that beneficiaries have bed nets and widely report the use of them. Beneficiaries recognized the role of community actors (teachers, community health workers, religious leaders) in dissemination of malaria key preventive messages. It was identified that there is room for improvement on SBC messaging regarding malaria symptoms. Specifically, that people recognize anaemia and convulsions as malaria symptoms so that they quickly seek health care services. Moreover, the study raises the need for further research into the main drivers of malaria prevalence in the country and the need to conduct operational research to refine SBC approaches.

## Data Availability

The datasets used and/or analysed during the current study are available from the corresponding author upon reasonable request.

## Abbreviations

HH: Household
ITNs: Insecticide-treated nets
SBC: Social and behavioural change
UCC: Universal coverage campaign
ANC: Antenatal care
NMCP: National Malaria Control Programme
ITN: Insecticide-treated net
IRS: Indoor residual spraying
KAP: Knowledge, attitude and practice
RDT: Rapid diagnostic test
